# Betulinic Acid Protects from Ischemia-Reperfusion Injury in the Mouse Retina

**DOI:** 10.3390/cells10092440

**Published:** 2021-09-16

**Authors:** Aytan Musayeva, Johanna C. Unkrig, Mayagozel B. Zhutdieva, Caroline Manicam, Yue Ruan, Panagiotis Laspas, Panagiotis Chronopoulos, Marie L. Göbel, Norbert Pfeiffer, Christoph Brochhausen, Andreas Daiber, Matthias Oelze, Huige Li, Ning Xia, Adrian Gericke

**Affiliations:** 1 Department of Ophthalmology, University Medical Center, Johannes Gutenberg University Mainz, Langenbeckstrasse 1, 55131 Mainz, Germany; aytan_musayeva@meei.harvard.edu (A.M.); charlotte.unkrig@gmail.com (J.C.U.); dr.zhutdieva@mail.ru (M.B.Z.); caroline.manicam@unimedizin-mainz.de (C.M.); yruan@uni-mainz.de (Y.R.); panagiotis.laspas@unimedizin-mainz.de (P.L.); panagiotis.chronopoulos@hotmail.com (P.C.); malu.goebel@yahoo.de (M.L.G.); norbert.pfeiffer@unimedizin-mainz.de (N.P.); 2 Laboratory of Corneal Immunology, Transplantation and Regeneration, Schepens Eye Research Institute, Massachusetts Eye and Ear, Department of Ophthalmology, Harvard Medical School, Boston, MA 02114, USA; 3 Institute of Pathology, University Medical Center, Johannes Gutenberg University Mainz, Langenbeckstrasse 1, 55131 Mainz, Germany; christoph.brochhausen@ukr.de; 4 Institute of Pathology, University of Regensburg, Franz-Josef-Strauß-Allee 11, 93053 Regensburg, Germany; 5 Department of Cardiology 1, Laboratory of Molecular Cardiology, University Medical Center, Johannes Gutenberg University Mainz, Building 605, Langenbeckstrasse 1, 55131 Mainz, Germany; daiber@uni-mainz.de (A.D.); Matthias.oelze@unimedizin-mainz.de (M.O.); 6 Department of Pharmacology, University Medical Center, Johannes Gutenberg University Mainz, Langenbeckstrasse 1, 55131 Mainz, Germany; huigeli@uni-mainz.de (H.L.); xianing@uni-mainz.de (N.X.)

**Keywords:** arterioles, betulinic acid, ischemia-reperfusion injury, reactive oxygen species, retina

## Abstract

Ischemia/reperfusion (I/R) events are involved in the pathophysiology of numerous ocular diseases. The purpose of this study was to test the hypothesis that betulinic acid protects from I/R injury in the mouse retina. Ocular ischemia was induced in mice by increasing intraocular pressure (IOP) to 110 mm Hg for 45 min, while the fellow eye served as a control. One group of mice received betulinic acid (50 mg/kg/day p.o. once daily) and the other group received the vehicle solution only. Eight days after the I/R event, the animals were killed and the retinal wholemounts and optic nerve cross-sections were prepared and stained with cresyl blue or toluidine blue, respectively, to count cells in the ganglion cell layer (GCL) of the retina and axons in the optic nerve. Retinal arteriole responses were measured in isolated retinas by video microscopy. The levels of reactive oxygen species (ROS) were assessed in retinal cryosections and redox gene expression was determined in isolated retinas by quantitative PCR. I/R markedly reduced cell number in the GCL and axon number in the optic nerve of the vehicle-treated mice. In contrast, only a negligible reduction in cell and axon number was observed following I/R in the betulinic acid-treated mice. Endothelial function was markedly reduced and ROS levels were increased in retinal arterioles of vehicle-exposed eyes following I/R, whereas betulinic acid partially prevented vascular endothelial dysfunction and ROS formation. Moreover, betulinic acid boosted mRNA expression for the antioxidant enzymes SOD3 and HO-1 following I/R. Our data provide evidence that betulinic acid protects from I/R injury in the mouse retina. Improvement of vascular endothelial function and the reduction in ROS levels appear to contribute to the neuroprotective effect.

## 1. Introduction

Ischemia/reperfusion (I/R) events have been implicated in the pathophysiology of various retinal diseases, such as retinal vascular occlusion, diabetic retinopathy and glaucoma [1]. Especially acute forms of vascular occlusion, such as central retinal artery occlusion (CRAO) are known to have a deleterious impact on visual acuity after an already short period of time [2]. The lack of oxygen supply to the retina often results in visual impairment and additional sequelae, such as retinal or vitreous hemorrhage, retinal neovascularization or neovascular glaucoma [3]. Arterial fibrinolysis failed to improve the clinical outcome of CRAO compared to conservative treatment, such as the application of acetylsalicylic acid and ocular massage, suggesting that deleterious, yet poorly understood, molecular processes are already activated in the early phase of retinal ischemia [4,5]. Hence, therapeutic approaches aimed at improving the resistance of retinal cells to I/R events are needed. We and others have previously demonstrated that oxidative stress plays a crucial role in mediating retinal tissue damage under hypoxic conditions and following I/R events [6,7,8,9].

The pentacyclic triterpenoid, betulinic acid, can be found in the peel of fruits, in leaves and in the stem bark of various plants, such as the white birch [10]. Initially, betulinic acid was found to exhibit biological activity against lymphocytic leukemia but was later found to exert effects against a variety of other tumors [11,12]. Moreover, the substance was reported to have anti-inflammatory, antiviral, antibacterial, antimalarial and antioxidant properties [13,14].

Recent studies have shown that betulinic acid protects against myocardial, renal and cerebral I/R injury [15,16,17,18,19]. However, the effects of betulinic acid on I/R are unknown in the retina; thus, the purpose of the present study was to test the hypothesis that betulinic acid protects from I/R injury in the mouse retina. Another goal of the study was to examine the involvement of oxidative stress in this process.

For our studies, we used a model in which intraocular pressure (IOP) was elevated by cannulation of the anterior chamber and administration of normal saline under high pressure, which leads to complete occlusion of blood vessels by compression [20,21].

## 2. Materials and Methods

### 2.1. Animals

All animal experiments were performed in accordance with the EU Directive 2010/63/EU for animal experiments and were approved by the Animal Care Committee of Rhineland-Palatinate, Germany (approval number: 23 177-07/G 13-1-064). Experiments were performed in 6-month-old, male C57Bl/6J mice. Mice were housed under standardized conditions with a 12 h light/dark cycle, a temperature of 22 ± 2 °C, humidity of 55 ± 10% and with free access to food and tap water.

### 2.2. Application of Betulinic Acid and Induction of Ischemia-Reperfusion Injury

One day before induction of I/R, mice received either betulinic acid (BioSolutions Halle GmbH, Halle, Germany) at 50 mg/kg body weight diluted in dimethyl sulfoxide (DMSO, Carl Roth GmbH, Karlsruhe, Germany) or DMSO (vehicle solution) via gavage. Twenty-four hours later, mice received a second dose of betulinic acid or vehicle solution and were subsequently anesthetized with xylocaine (1 mg/mL, i.p.) and ketamine (10 mg/mL, i.p.). Body temperature was kept constant at 37 °C using a heating pad. Retinal ischemia was induced in a randomly chosen eye by introducing the tip of a glass micropipette (100 µm diameter) into the anterior chamber. The micropipette was attached via a silicon tube to a saline-filled (0.9% NaCl) reservoir that was raised above the mouse to increase intraocular pressure (IOP) to 110 mm Hg for 45 min. The fellow eye, which served as a control, was also cannulated in the same manner and maintained at an IOP of 15 mm Hg for 45 min. Retinal ischemia was considered complete when whitening of the anterior segment of the eye was observed by microscopic examination. Ofloxacin ophthalmic ointment (3 mg/g, Bausch + Lomb, Berlin, Germany) was applied on the ocular surface after needle removal. For the following seven days, mice received either betulinic acid or the vehicle solution once daily. Eight days after the I/R event, mice were sacrificed for further studies.

### 2.3. Retinal Wholemounts and Cell Counting

After mice had been sacrificed by CO_2_ inhalation, the eye globes were removed using fine-point tweezers and Vannas scissors and fixed in 4% phosphate-buffered paraformaldehyde (Sigma-Aldrich, Munich, Germany) for one hour. Then, retinas were isolated from the eye globes in phosphate-buffered solution (PBS, Invitrogen, Karlsruhe, Germany) by using fine-point tweezers and Vannas scissors. After isolation, wholemounts were prepared and stained with cresyl blue using a standard protocol [22]. After de- and rehydration using increasing and decreasing concentrations of ethanol (70–100%), wholemounts were placed in distilled water and stained with 2% cresyl blue (Merck, Darmstadt, Germany). Next, wholemounts were dehydrated in ethanol, incubated in xylene and embedded in a quick-hardening mounting medium (Eukitt, Sigma-Aldrich). Subsequently, wholemounts were viewed under a light microscope (Vanox-T, Olympus, Hamburg, Germany) connected to a Hitachi CCD camera (Hitachi, Düsseldorf, Germany) and equipped with Diskus software (Carl H. Hilgers, Königswinter, Germany). Per wholemount, 16 pre-defined areas, eight central and eight peripheral, of 150 µm × 200 µm were photographed by a blinded investigator as previously described [22]. The proximal border of a central area was localized 0.75 mm from the center of the papilla. This distance corresponded to 5 heights of a photographed area. Each proximal border of a peripheral area was localized 0.75 mm from the distal border of a central photographed area. Thus, the distance from the center of the papilla and the proximal border of a peripheral area was 1.65 mm. In each photograph, cells were counted manually using the cell counter plug-in for ImageJ software (NIH, http://rsb.info.nih.gov/ij/) accessed on 11 March 2019. The mean cell density was calculated and the total number of cells per retina was assessed by multiplying the mean density by the area of the wholemount.

### 2.4. Optic Nerve Cross-Sections and Axon Counting

Optic nerves were dissected and placed in a fixative solution (2.5% glutaraldehyde and 2.0% paraformaldehyde in 0.15 M cacodylate buffer). Later, nerve segments were postfixed in 1% osmium tetroxide, dehydrated in ethanol and acetone, stained in 2% uranyl acetate, embedded in agar 100 resin (PLANO, Wetzlar, Germany) and submitted to polymerization at 60 °C for at least 48 h, according to standard protocols. Next, semithin cross-sections were cut with an ultramicrotome (Ultracut E, Leica, Bensheim, Germany), placed on conventional glass slides and stained with 1% toluidine blue in 1% sodium borate. Microscopical analysis and photomicroscopy of the cross-sections were performed with a light microscope (Vanox-T, Olympus) by a blinded investigator. The whole surface of each cross-section was assessed microscopically. Five non-overlapping fields of 80 µm × 60 µm (one central and four in the periphery) were photographed (Hitachi CCD camera) on every cross-section as previously described [22]. The axons were counted manually on these photographs using ImageJ software. The mean axon density was calculated and the total number of axons per optic nerve was assessed by multiplying the mean density by the cross-sectional area.

### 2.5. Measurements of Retinal Arteriole Reactivity

Retinal arteriole reactivity was measured in isolated retinas using video microscopy as previously described [23,24]. First, mice were sacrificed by CO_2_ exposure and one eye per mouse was isolated and transferred into cold Krebs-Henseleit buffer. After preparation of the ophthalmic artery and isolation of the retina, the ophthalmic artery was canulated and the retina placed onto a transparent plastic platform. Next, retinal arterioles were pressurized by raising a reservoir connected to the micropipette to a level corresponding to 50 mmHg. Then, first-order retinal arterioles were imaged under brightfield conditions. After an equilibration period of 30 min, concentration-response curves for the thromboxane mimetic, U46619 (10^−11^ to 10^−6^ M; Cayman Chemical, Ann Arbor, MI, USA), were conducted. The arterioles were then preconstricted to 50–70% of the initial luminal diameter by titration of U46619, and responses to the endothelium-dependent vasodilator acetylcholine (10^−9^ to 10^−4^ M; Sigma-Aldrich, Taufkirchen, Germany) and the endothelium-independent nitric oxide (NO) donor, sodium nitroprusside (SNP, 10^−9^ to 10^−4^ M; Sigma-Aldrich), were measured.

### 2.6. Assessment of ROS Levels

The fluorescent dye, dihydroethidium (DHE), was used to determine ROS levels in situ as described previously [25,26]. After mice had been sacrificed and their eyes harvested, frozen cross-sections of 10 µm thickness were prepared. After thawing, the tissue sections were immediately incubated with 1 µM of dihydroethidium (DHE, Thermo Fischer Scientific, Waltham, MA, USA). DHE is cell-permeable and reacts with superoxide to form ethidium, which in turn intercalates in deoxyribonucleic acid, thereby exhibiting red fluorescence. Using an Eclipse TS 100 microscope (Nikon, Tokyo, Japan) equipped with a DS–Fi1-U2 digital microscope camera (Nikon, Tokyo, Japan) and the imaging software NIS Elements (Version 1.10, Nikon, Tokyo, Japan) the fluorescence (518 nm/605 nm excitation/emission) was recorded and measured in retinal cross-sections by using ImageJ.

### 2.7. Quantitative PCR Analysis

Messenger RNA for the hypoxic markers, hypoxia-inducible factor 1α (HIF-1α) and vascular endothelial growth factor-A (VEGF-A), the prooxidant isoforms of the nicotinamide adenine dinucleotide phosphate oxidase, NOX1, NOX2 and NOX4, the antioxidant redox enzymes, catalase (CAT), glutathione peroxidase 1 (GPX1), heme oxygenase 1 (HO-1), the three isoforms of superoxide dismutase (SOD), SOD1, SOD2 and SOD3 and for the three nitric oxide synthase (NOS) isoforms, eNOS, iNOS and nNOS, was quantified in the retina by quantitative PCR (qPCR). After mice had been killed by CO_2_ inhalation, one eye per mouse was immediately excised and transferred into cooled PBS (Invitrogen, Karlsruhe, Germany). Next, the retina was isolated by Vannas scissors and fine-point tweezers, transferred into a 1.5 mL plastic tube, rapidly frozen in liquid nitrogen and stored at −80 °C. Within 3 months, tissue samples were homogenized (FastPrep, MP Biomedicals, Illkirch, France) and the expression of genes was measured by SYBR Green-based quantitative real-time PCR, as previously described [27]. RNA was isolated using peqGOLD TriFast™ (PEQLAB) and cDNA was generated with the High Capacity cDNA Reverse Transcription Kit (Applied Biosystems, Darmstadt, Germany). Real-time PCR reactions were performed on a StepOnePlus™ Real-Time PCR System (Applied Biosystems) using SYBR^®^ Green JumpStart™ Taq ReadyMix™ (Sigma-Aldrich) and 20 ng cDNA. The relative mRNA levels of the target genes were quantified using comparative threshold (CT) normalized to the TATA-binding protein (TBP) housekeeping gene. Messenger RNA expression is presented as the fold-change to vehicle-treated eyes. The PCR primer sequences are listed in Table 1.

### 2.8. Statistical Analysis

Data are presented as the mean ± SE and n represents the number of mice per group. For the comparison of cell numbers, axon numbers, DHE staining intensity and mRNA expression levels, a one-way ANOVA and the Tukey’s multiple comparisons test were used. Vasoconstrictor responses to U46619 are presented as percent change in luminal diameter from resting diameter, whereas responses to acetylcholine and SNP are presented as percent change in luminal diameter from preconstricted diameter. The comparison between concentration-responses was made using a two-way ANOVA for repeated measurements and the Tukey’s multiple comparisons test. The level of significance was set at 0.05.

## 3. Results

### 3.1. Number of Cells in the Retinal Ganglion Cell Layer and of Axons in the Optic Nerve

**Figure 1:**
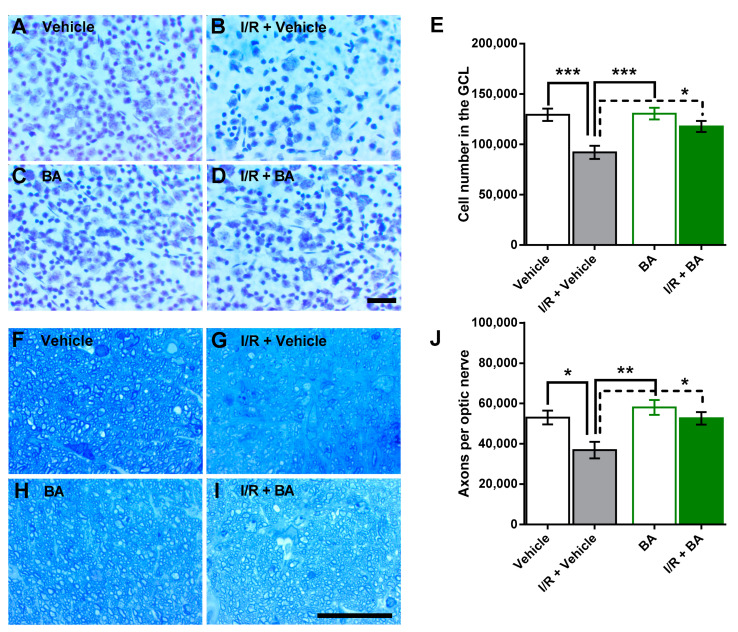
Total cell number in the ganglion cell layer (GCL) of the retina and axon number in the optic nerve. (**A**–**D**) Representative pictures of cells in the GCL stained with cresyl blue. Scale bar = 30 µm. (**E**) I/R markedly reduced the total cell number in the GCL in vehicle-treated mice but not in betulinic acid (BA)-treated mice (*** *p* < 0.001; * *p* < 0.05; *n* = 8 per group). (**F**–**I**) Representative pictures of optic nerve axons stained with toluidine blue. Scale bar = 30 µm. (**J**) I/R reduced axon number in the optic nerve in vehicle-treated mice but not in BA-treated mice (** *p* < 0.01; * *p* < 0.05; *n* = 8 per group).

Ischemia-reperfusion markedly reduced the cell number in the retinal ganglion cell layer of vehicle-treated mice. Total cell number in the retinal ganglion cell layer was 129,378 ± 6103 cells and 92,053 ± 6580 cells in retinas from vehicle-treated and I/R + vehicle-treated eyes, respectively (***p < 0.001), which constitutes a reduction of ≈29% following I/R. In contrast, only a negligible reduction of ≈10% in cell number was observed in the betulinic acid-treated eyes (130,468 ± 5791 versus 117,836 ± 5504, betulinic acid versus I/R + betulinic acid-treated eyes, *p* > 0.05) (Figure 1A–E). Cells in the mouse retinal ganglion cell layer are mainly comprised of neurons, but also vascular endothelial cells and glial cells [22,28]. The neurons are composed primarily of retinal ganglion cells and displaced amacrine cells. Notably, retinal ganglion cells account for only about half of the neurons in the retinal ganglion cell layer of the mouse eye [22,29]. Since the cresyl blue staining method does not clearly distinguish between ganglion cells and other neurons because of some overlap in nuclear size and shape, we also calculated the axons of retinal ganglion cells in optic nerve cross-sections. Of note, I/R also reduced the number of optic nerve axons in the vehicle-treated mice. Axon number was 52,994 ± 3411 and 36,796 ± 4079 in vehicle-treated versus I/R + vehicle-treated eyes (*p < 0.05), which is a reduction of ≈31% following I/R. In contrast, I/R had only a negligible effect (reduction of ≈9%) on optic nerve axon number in the betulinic acid-treated mice (58,019 ± 3674 and 52,603 ± 3111, betulinic acid versus I/R + betulinic acid, *p* > 0.05) (Figure 1F–J).

### 3.2. Retinal Arteriole Responses

**Figure 2:**
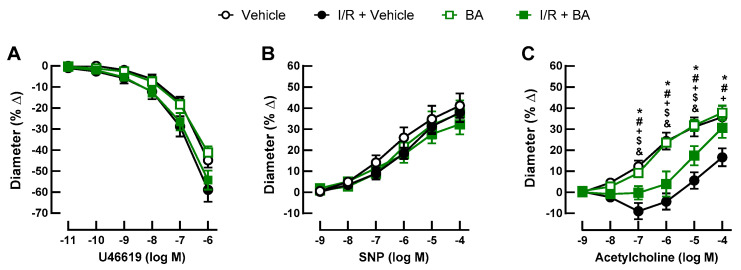
Responses of retinal arterioles to vasoactive substances. (**A**) The thromboxane mimetic, U46619, elicited concentration-dependent vasoconstriction in retinal arterioles that was similar in all groups. (**B**) Likewise, responses to the endothelium-independent vasodilator, sodium nitroprusside (SNP), did not differ between the four groups. (**C**) In contrast, retinal arterioles from mice subjected to I/R displayed blunted endothelium-dependent vasodilator responses to acetylcholine, which were partially improved by treatment with BA. Values are expressed as the mean ± SE (* *p* < 0.05, I/R + vehicle versus vehicle; # *p* < 0.05, I/R + vehicle versus BA; + *p* < 0.05, I/R + vehicle versus I/R + BA; $ *p* < 0.05, I/R + BA versus vehicle; & *p* < 0.05, I/R + BA versus BA; n = 8 per group).

U46619 (10^−11^–10^−6^ M) elicited concentration-dependent vasoconstriction of retinal arterioles that was similar in all groups (Figure 2A). Likewise, endothelium-independent vasodilation induced by SNP (10^−9^–10^−4^ M) was similar in all four groups (Figure 2B). In contrast, acetylcholine-induced (10^−9^–10^−4^ M) vasodilation was greatly reduced in the arteries of mice exposed to I/R and the vehicle (Figure 2C). Of note, betulinic acid partially prevented endothelial dysfunction following I/R (Figure 2C).

### 3.3. ROS Levels in the Retina

**Figure 3:**
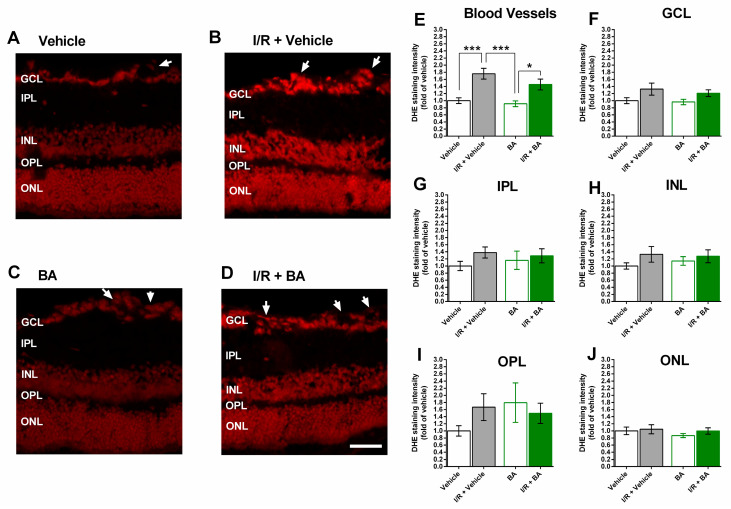
Dihydroethidium (DHE) staining in retinal cross-sections. (**A**–**D**) Representative pictures of retinal cross-sections from each group. Scale bar = 50 µm. (**E**–**J**) DHE staining intensity was markedly increased in retinal blood vessels from I/R- and vehicle-treated eyes (**E**). In none of the retinal layers, marked differences in DHE staining intensity were observed among groups (**F**–**J**) (GCL, ganglion cell layer; IPL, inner plexiform layer; INL, inner nuclear layer; OPL, outer plexiform layer; ONL, outer nuclear layer; *** *p* < 0.001; * *p* < 0.05; *n* = 8 per group).

The staining of retinal cross-sections with DHE revealed markedly increased staining intensity in retinal blood vessels from eyes exposed to I/R and the vehicle (Figure 3A–E), indicative of increased vascular ROS concentration. In contrast, DHE staining intensity did not differ between the four groups in any of the retinal layers (Figure 3F–J).

### 3.4. Messenger RNA Expression in the Retina

**Figure 4:**
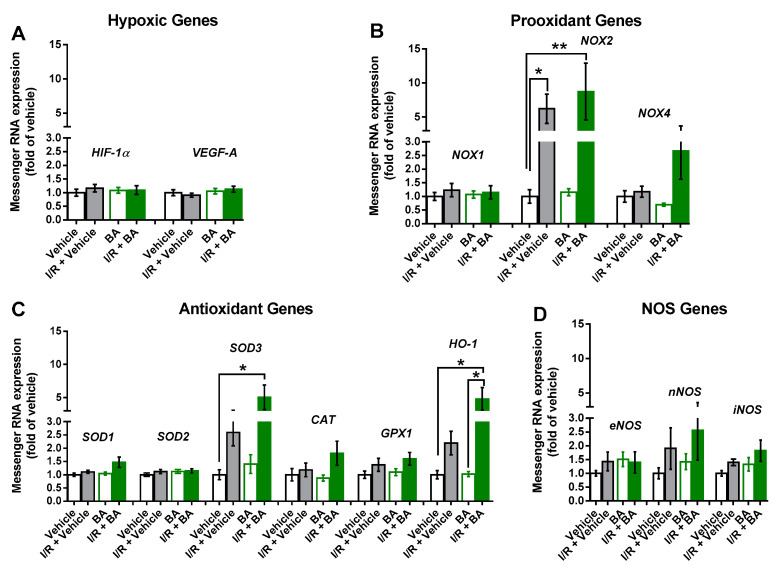
Messenger RNA expression of the hypoxic genes, *HIF-1α* and *VEGF-A* (**A**), the prooxidant genes (*NOX1*, *NOX2*, *NOX4*) (**B**), the antioxidant genes (*SOD1*, *SOD2*, *SOD3*, *CAT*, *GPX1*, *HO-1*) (**C**) and of the NOS genes (*eNOS*, *nNOS*, *iNOS*) (**D**) in the eyes treated with vehicle only, I/R + vehicle, betulinic acid (BA) only and I/R + BA. Notably, BA did not prevent upregulation of *NOX2* expression induced by I/R. However, mRNA expression of the antioxidant redox genes, *SOD3* and *HO-1*, was markedly increased in mice exposed to I/R and BA. Data are presented as the mean ± SE (** *p*  <  0.01; * *p*  <  0.05; *n*  =  8 per group).

Notably, mRNA for the hypoxic genes, HIF-1α and VEGF-A, was not elevated following I/R (Figure 4A). In contrast, mRNA for NOX2 was elevated to a similar extent following I/R in the vehicle-exposed and betulinic acid-exposed mice (Figure 4B), suggesting that betulinic acid had no effect on I/R-induced NOX2 mRNA expression. Remarkably, betulinic acid boosted retinal mRNA expression for the antioxidant enzymes, SOD3 and HO-1 (Figure 4C) but had no effect on NOS mRNA expression (Figure 4D).

## 4. Discussion

There are several major new findings in the present study. First, following I/R, betulinic acid prevented cell loss in the retinal GCL and axon loss in the optic nerve, indicative of a protective effect on retinal ganglion cells. Second, I/R induced endothelial dysfunction in retinal arterioles, which was partially prevented by betulinic acid. Third, betulinic acid reduced the generation of ROS in retinal vessels following I/R. Fourth, treatment with betulinic acid-enhanced mRNA expression for the antioxidant enzymes, SOD3 and HO-1, while it did not prevent an increase in mRNA levels for the prooxidant NADPH oxidase subunit, NOX2, following I/R.

This is the first study to report on a protective effect of betulinic acid on I/R injury in the retina. Several previous studies reported on the protective effects of betulinic acid in I/R models of other organs. For example, in an ischemic heart model in which rats were pretreated for 7 days with betulinic acid (50, 100 and 200 mg/kg, i.g.) before cardiac ischemia was induced by 30 min of left anterior descending artery occlusion followed by 2 h of reperfusion, betulinic acid improved left ventricular function, suppressed myocardial apoptosis and reduced the release of lactate dehydrogenase and creatine kinase [16]. In a rat renal I/R model, the renal pedicle was occluded for 45 min to induce ischemia followed by reperfusion for 6 h. Rats that were treated with betulinic acid (250 mg/kg, i.p.) on two occasions, 30 min prior to ischemia and immediately before the reperfusion period, had attenuated I/R-induced oxidant responses, reduced microscopic damage and better renal function [17]. Likewise, a study in the rat brain reported that pretreatment with betulinic acid for seven days at 50 mg/kg i.g. reduced cerebral injury and oxidative stress after one hour of middle cerebral artery occlusion followed by 24 h of reperfusion by activation of the SIRT1/FoxO1 pathway and the suppression of autophagy [19]. Similarly, mouse brain pretreatment with betulinic acid for seven days at 50 mg/kg/day p.o. reduced I/R-induced infarct volume and ameliorated the neurological deficit after two hours of middle cerebral artery occlusion followed by 22 h of reperfusion in hypercholesterolemic apolipoprotein E knockout mice. This was accompanied by the prevention of NOX2, nNOS and iNOS upregulation and attenuation of oxidative stress [15]. Another study in the mouse brain reported that betulinic acid reduced ROS production together with mRNA levels for NOX4 following I/R [18].

Our study is in line with the previously reported observations in other organs by demonstrating that betulinic acid protects retinal cells from I/R-induced damage. Since only around half of the cells in the retinal GCL are actually retinal ganglion cells [29], a cell subgroup that transmits visual information from the retina to the brain, we calculated the axons of retinal ganglion cells in optic nerve cross-sections in order to specify its number. Of note, betulinic acid protected retinal ganglion cells from I/R injury.

The present study extends the previously reported observations by demonstrating that vascular endothelial function was impaired one week after the I/R event and that betulinic acid reduced the extent of endothelial dysfunction. We have previously shown in a pig model of ocular ischemia that retinal endothelial function was impaired after only 12 min of ischemia followed by 20 h of reperfusion [6]. The present study suggests that the vascular endothelial recovery is not finished one week after the I/R event. This finding may be important for at least a subgroup of patients with glaucoma, who seem to be predisposed to repeated I/R events of the retina and optic nerve [30,31,32]. Based on these findings, an acute IOP increase may cause sustained vascular dysfunction, which in turn may itself predispose to further I/R events resulting in damage of retinal ganglion cells.

Remarkably, betulinic acid ameliorated I/R-induced vascular endothelial dysfunction in retinal arterioles. Vasoprotective effects have already been reported for betulinic acid in larger blood vessels. In these studies, exposure to betulinic acid improved endothelial function and reduced vascular ROS levels [33,34,35].

Although we found positive effects of betulinic acid on neuron survival and retinal endothelial function, we did not find direct effects of betulinic acid on oxidative stress. However, our study protocol differed from protocols in the previously reported studies in several aspects. First, we started administration of betulinic acid one day prior to the I/R event and continued application until the seventh day after the event. We did so because the full extent of retinal neuronal damage is not visible directly after the I/R event. Second, we measured vascular reactivity, ROS levels and mRNA expression at one time point eight days after the I/R event. This may be the reason why we found ROS levels to be elevated significantly only in retinal blood vessels, but not in individual retinal layers. The oxidative stress, which was observed in the retina in the acute phase after I/R events in various studies, including our own, may return to normal after several days [6,8]. In support of this hypothesis, a study in mice that utilized the chemiluminescent probe, L-012, as a noninvasive in vivo ROS detection agent demonstrated that ROS levels were tremendously increased one day after the I/R event while they were already markedly lower after three and seven days [36].

However, we found indirect hints that an oxidative burst occurred following I/R, because mRNA for the prooxidant NADPH oxidase subunit, NOX2, was elevated eight days following I/R. We and others have previously reported that NOX2 mRNA and protein levels were elevated following I/R in the retina of various species, including mice and pigs [6,8]. Since in the present study, NOX2 mRNA levels were similarly elevated in the I/R + vehicle group and in the I/R + betulinic acid group, betulinic acid apparently had no major effects on NOX2 mRNA expression. We also did not find evidence for the downregulation of NOX4 mRNA expression by betulinic acid as previously suggested in the mouse brain [18].

However, we found that betulinic acid-enhanced mRNA expression for the antioxidant enzymes SOD3 and HO-1, which have both previously been demonstrated to exert antioxidant effects in the retina. For example, SOD3 was shown to reduce oxidative stress in the inner retina and at the vitreoretinal interface in mice [37]. Similarly, HO-1 was shown to exert potent antioxidant, antiapoptotic, anti-inflammatory and cytoprotective activities against I/R injury in various organs, including the retina [38].

One potential limitation of this study is that the samples for quantification of redox gene mRNA and oxidative stress were taken eight days after the I/R event, which may be too long to detect acute changes in oxidative stress and redox gene expression in response to I/R. Hence, the choice of this time point may underestimate the contribution of ROS and some redox genes to ischemic injury or to neuro- and vasoprotection. On the other hand, the choice of this time point gives us a picture of prolonged molecular changes following I/R. Moreover, in the present study, mice received betulinic acid one day before I/R and continued receiving the substance for seven days after the event because the aim of the study was to determine whether betulinic acid exerted neuroprotective properties at all. It remains to be established whether betulinic acid can protect from I/R when its administration is started after the I/R event, a situation typically seen in a clinical setting when patients come to the ophthalmologist after they experience visual problems due to an I/R event.

## 5. Conclusions

In conclusion, this is the first study demonstrating protective effects of betulinic acid following I/R in the retina, which is in line with previous studies in other organs, such as the brain, heart and kidney. Another new finding is that vascular endothelial function was markedly impaired eight days after the retinal I/R event, which suggests that even short periods of I/R, as observed in acute IOP increases, may lead to sustained functional deficits of the retinal vasculature. Remarkably, betulinic acid partially prevented endothelial dysfunction following I/R. From a clinical point of view, betulinic acid may become useful in treating ischemic diseases of the retina and optic nerve.

## Figures and Tables

**Table 1:** Primer sequences used for quantitative PCR analysis.

Gene	Forward	Reverse
*NOX1*	GGAGGAATTAGGCAAAATGGATT	GCTGCATGACCAGCAATGTT
*NOX2*	CCAACTGGGATAACGAGTTCA	GAGAGTTTCAGCCAAGGCTTC
*NOX4*	TGTAACAGAGGGAAAACAGTTGGA	GTTCCGGTTACTCAAACTATGAAGAGT
*eNOS*	CCTTCCGCTACCAGCCAGA	CAGAGATCTTCACTGCATTGGCTA
*iNOS*	CAGCTGGGCTGTACAAACCTT	CATTGGAAGTGAAGCGTTTCG
*nNOS*	TCCACCTGCCTCGAAACC	TTGTCGCTGTTGCCAAAAAC
*CAT*	CAAGTACAACGCTGAGAAGCCTAAG	CCCTTCGCAGCCATGTG
*GPX1*	CCCGTGCGCAGGTACAG	GGGACAGCAGGGTTTCTATGTC
*HO-1*	GGTGATGCTGACAGAGGAACAC	TAGCAGGCCTCTGACGAAGTG
*SOD1*	CCAGTGCAGGACCTCATTTTAAT	TCTCCAACATGCCTCTCTTCATC
*SOD2*	CCTGCTCTAATCAGGACCCATT	CGTGCTCCCACACGTCAAT
*SOD3*	TTCTTGTTCTACGGCTTGCTACTG	AGCTGGACTCCCCTGGATTT
*TBP*	CTTCGTGCAAGAAATGCTGAAT	CAGTTGTCCGTGGCTCTCTTATT

## Data Availability

The data presented in this study are available on request from the corresponding author.
